# Alpha_1A_-adrenoceptor antagonist improves underactive bladder associated with diabetic cystopathy via bladder blood flow in rats

**DOI:** 10.1186/s12894-017-0256-9

**Published:** 2017-08-23

**Authors:** Saori Yonekubo, Satoshi Tatemichi, Kazuyasu Maruyama, Mamoru Kobayashi

**Affiliations:** Central Research Laboratories, Kissei Pharmaceutical Co., Ltd, 4365-1 Kashiwabara, Hotaka, Azumino-City, Nagano-Pref 399-8304 Japan

**Keywords:** Alpha-adrenergic receptor, Blood flow, Diabetes, Underactive bladder

## Abstract

**Background:**

Patients with diabetes experience lower urinary tract symptoms. Cystopathy may evolve into underactive bladder (UAB), depending on the degree and duration of the symptoms. In the present study, we aimed to investigate the effects of silodosin, an alpha_1A_-adrenoceptor (AR) antagonist, on UAB in a rat model of diabetes mellitus (DM).

**Methods:**

Female Sprague-Dawley rats (6 weeks old) were administered streptozotocin (STZ) (50 mg/kg, i.v.) to establish a DM model. One week after STZ administration, vehicle or silodosin (0.3 or 1 mg/kg/day) was delivered subcutaneously through an osmotic pump. Nine weeks after STZ administration (8 weeks after drug treatment), a catheter was implanted into the bladder under urethane anesthesia. After the measurement of emptied bladder blood flow (BBF), saline was continuously infused into the bladder and intravesical pressure and micturition volume were measured. In another experiment, the bladder was isolated and nerve markers were quantified.

**Results:**

A cystometrogram showed that bladder capacity (BC), residual volume (RV), and bladder extension (BC/bladder weight) increased by 7.43, 10.47, and 3.59 times, respectively, in vehicle rats in comparison with normal rats. These findings suggested the occurrence of UAB-like symptoms in this model. Silodosin (1 mg/kg/day) inhibited the increase in BC and RV by 49.0% and 46.8%, respectively, and caused a decrease in BBF of approximately 25.5% (when the difference between normal and vehicle was set as 100%) in STZ rats. The nerve marker expression levels tended to be decreased in the bladders of STZ rats and these effects were ameliorated by silodosin.

**Conclusions:**

The STZ rats showed increased bladder extension and RV, symptoms that were suggestive of UAB, and these symptoms were ameliorated by silodosin. These results suggested that the alpha_1A_-AR antagonist would be useful for the prevention or treatment of UAB.

## Background

More than 371 million people worldwide suffered from diabetes in 2012 [1]. Various complications are associated with diabetes. Over 50% of diabetic patients have diabetic cystopathy, such as overactive bladder (OAB) syndrome and incontinence [2, 3]. In diabetic patients with lower urinary tract symptoms (LUTS), OAB may evolve into underactive bladder (UAB), or detrusor underactivity (DUA), depending on the degree and duration of the symptoms. Diabetic UAB is characterized by an impaired sensation of bladder fullness, increased bladder capacity (BC), decreased bladder contractility, and increased post voiding residual volume (RV) [4, 5]. When urinary symptoms in patients become severe, they may develop into incontinence, ischuria, and/or hydronephrosis. OAB became a common disease, whereas UAB has not been studied in detail [4].

Diabetes mellitus (DM), bladder outlet obstruction (BOO), and aging are considered factors contributing to the development of UAB symptoms. Rat models of DM, BOO, aging, and pelvic nerve transection have been reported previously [4, 6]. Changes in parameters suggesting the functional decline of the bladder have been observed in all models. DM rats have DUA, hypoesthesia, and a large RV. Bladder nerves and vessels have been reported to show decreased density in DM rat models [7–9]. Diabetes is known to induce functional changes related to neuropathy, and blood flow is thought to be closely related to the nerves. However, there have been few detailed reports regarding the nerve changes associated with bladder blood flow (BBF) in DM models.

The pharmacological treatment options for UAB syndrome are limited. This condition can be improved with the use of agents that increase detrusor contractile activity and/or decrease outlet resistance. Current standard pharmacotherapy includes the use of muscarinic receptor agonists, such as bethanechol, to stimulate detrusor muscarinic receptors, or cholinesterase inhibitors, such as distigmine, to reduce the degradation of acetylcholine [10]. Alpha_1_-Adrenoceptor (AR) antagonists have also been reported to be an used for treatment for UAB [11–13]. The mechanism of action of alpha_1_-AR antagonists involves a cancellation of confinement through the inhibition of alpha_1A_-ARs distributed mainly over the urethra (prostatic part) [14]. Urapidil is used for relaxing the urethra and reducing resistance to urine flow and is the only alpha_1_-AR antagonist that can be used in women [11]. However, hypotension develops concomitantly with urethral relaxation because urapidil has a lower selectivity for alpha_1A_-ARs [15, 16] than for alpha_1B_-ARs, which are mainly involved in blood pressure regulation [17]. Recent studies showed that chronic treatment with tamsulosin (alpha_1A/1D_–AR antagonist) prevented a decrease in BBF and controlled the increase in urinary frequency in rats [18, 19]. Furthermore, chronic treatment with silodosin (alpha_1A_-AR antagonist) reportedly improved bladder dysfunction by restoring the BBF in a rat model of atherosclerosis-induced bladder ischemia without BOO [20, 21]. These results suggest that alpha_1_-AR antagonists can not only relax urethral obstruction, which is their primary action (prostatic effect), but also improve BBF (bladder effect). Therefore, it is considered that alpha_1_-AR antagonists may improve BBF and ameliorate bladder dysfunction in addition to inducing urethral relaxation.

In this study, we investigated the effects of silodosin on the changes in bladder function in a streptozotocin (STZ)-induced DM rat model.

## Methods

### Animals

Female Sprague–Dawley rats (Charles River, Yokohama, Japan) were housed under a 12-h/12-h light cycle (lights on, 08:00–20:00 h) under controlled conditions and fed a laboratory chow diet and water ad libitum. All animal experiments were performed in accordance with the guidelines approved by the Laboratory Animal Committee of Kissei Pharmaceutical Co., Ltd., which conform to the current Japanese law.

### Induction of diabetes

In this study, diabetes was induced as reported previously [22]. Briefly, rats were injected with STZ dissolved in 0.03 mol/L citrate buffer at pH 4.5, which was used at a dose of 50 mg/kg (i.v.). Age-matched non-diabetic rats were injected with vehicle alone. After 1 week, non-fasting serum glucose levels were measured using Antsense III (Horiba, Kyoto, Japan), and rats with values above 300 mg/dL were considered diabetic. Rats were assigned based on body weight (BW) and blood glucose level. The rats that did not meet the experimental criterion were euthanized using the method of inhalation of carbon dioxide (CO_2_).

### Drug treatment

Drug treatment was performed using a procedure similar to that described previously [20]. When the blood glucose level was measured the following day, an osmotic pump (2ML4; Alzet, Cupertino, CA) was inserted under the dorsal skin. The silodosin group was subcutaneously administered the drug at each constant infusion rate for 8 weeks via the osmotic pump. Steady-state free plasma concentrations of silodosin were estimated using constant infusion equations, total clearance, and the percentage of silodosin binding to rat plasma proteins. Concentrations at the constant infusion rates of each dose were calculated as 1.13 × 10^−9^ mol/L (silodosin, 0.3 mg/kg/day) and 3.78 × 10^−9^ mol/L (silodosin, 1.0 mg/kg/day) [20]. Similarly, the sham treatment and DM groups were subcutaneously infused with Hartmann’s solution as the vehicle via the osmotic pump for 8 weeks.

### BBF

Measurements were performed using a procedure similar to that described previously [20]. Nine weeks after the induction of diabetes, the rats were anesthetized by the subcutaneous administration of urethane (1.0 g/kg). A midline abdominal incision was made to expose the anterior bladder. An 18-gauge catheter was implanted in the bladder through the dome and the bladder was emptied. BBF was determined using an Omegazone laser speckle blood flow imager (Omegawave, Tokyo, Japan), which shows blood flow as high-resolution 2-dimensional color images.

### Cystometrogram (CMG)

After the BBF study, cystometric measurements were performed. Briefly, the rat was placed in the supine position, and a bladder catheter was connected via a tube to a KDS-100 infusion pump (Muromachi Kikai, Tokyo, Japan) for continuous saline infusion into the bladder and a DT-4812 pressure transducer (Nihon Becton Dickinson, Tokyo, Japan) for bladder pressure recording. Micturition volume (MV) was measured using a GF-300 digital balance (A&D, Tokyo, Japan) located below the plastic cage. During cystometric evaluation, saline was infused to achieve a voiding interval of about 20 min (infusion rate, Normal rats: 1 ~ 3 mL/h, DM rats: 6 ~ 24 mL/h). Bladder pressure and MV were recorded continuously on a Rectigraph-8 K (NEC San-ei, Tokyo, Japan). The cystometric parameters obtained were intravesical pressure (IVP), MV, and RV. Rats used for CMG measurement were euthanized by exsanguination of the abdominal aorta.

### Real-time RT-PCR

Nine weeks after the induction of diabetes, total RNA in the bladder was extracted using an RNeasy® Fibrous Tissue Mini Kit (Qiagen, Hilden, Germany). For this experiment, we used different rats from those used for the blood flow measurement and CMG. Rats used for PCR and immunohistochemistry studies were euthanized by exsanguination of the abdominal aorta using isoflurane anesthesia. The mRNA expression levels corresponding to target genes were determined by real-time quantitative RT-PCR with an Applied Biosystems 7500 Fast (Life Technologies Japan, Tokyo, Japan) using a PrimeScript™ RT reagent kit (Takara, Shiga, Japan) and SYBR® Premix Ex Taq™ (Takara). The levels of β-actin, neurofilament-M (NF-M), and peripherin mRNA expression were quantified by the reverse-transcription of total bladder RNA using primers purchased from Takara. After extraction of total bladder RNA as described above, first-strand cDNA was synthesized from 0.4 mg of the total RNA using a GeneAmp PCR System 9700 (Life Technologies Japan) for RT-PCR. The total bladder RNA from normal rats was used to determine the standard curve. After correction for β-actin mRNA levels, the expression levels of the test genes were normalized to those in normal rats.

### Immunohistochemistry

The bladders were immersed in Mildform® (Wako, Osaka, Japan), embedded in paraffin, and cut into 5-μm-thick sections. Briefly, the sections were deparaffinized and then heated in a microwave oven for 30 min for antigen retrieval. After cooling and washing the sections with water, nonspecific binding of immunoglobulin was prevented using a protein block reagent (Agilent Technologies, Denmark, Denmark) for 10 min. After overnight incubation at 4 °C with anti-NF-M (AB1987, dilution 1:200; Merck KGaA, Darmstadt, Germany) or anti-peripherin (AB1530, dilution 1:100; Merck KGaA) antibody, the sections were rinsed with phosphate-buffered saline 3 times for 5 min each. The sections were then treated with Alexa Fluor® 488 goat anti-rabbit IgG (A11034, dilution 1:500; Life Technologies Japan) for 2 h at room temperature. After rinsing with phosphate-buffered saline 3 times for 5 min each, propidium iodide was added to stain the nuclei, followed by mounting with Fluoromount/Plus™ (Diagnostic BioSystems, Pleasanton, CA).

Photomicrographs (10×) of labeled bladder sections were taken using a DP72 digital camera (Olympus, Tokyo, Japan) attached to an Olympus IX71 microscope. The whole area of the bladder was photographed and tiled with high accuracy using the cellSens digital imaging software (Olympus), and WinROOF image processing, image measurement, image analysis, and data processing software (Mitani, Fukui, Japan) were used to measure the total area of the photographed bladder and immunopositive bundles.

### Drug

The test drug silodosin (KMD-3213; Kissei Pharmaceutical Co. Ltd., Matsumoto, Japan) was dissolved and diluted in Hartmann’s solution of the following composition (*w*/*v*%): 0.60 NaCl, 0.03 KCl, 0.02 CaCl_2_, and 0.31 lactic acid, containing hydrobromide at a 2-fold equivalent of silodosin.

### Data analysis

Data are presented as means ± S.E.M. Statistical analyses were performed using SAS version 8.20 (SAS Institute, Cary, NC). Data were analyzed among the 3 groups as follows. When equality of variances was indicated by Bartlett’s test, statistical analysis was performed using one-way analysis of variance (ANOVA) followed by parametric Dunnett’s multiple comparison test. When equality of variances was not indicated by Bartlett’s test, statistical analysis was performed using nonparametric Dunnett’s multiple comparison test. Comparisons between two groups were performed as follows. When equality of variances was indicated by an F-test, statistical analysis was performed using the Student’s t-test; when equality of variances was not indicated, statistical analysis was performed using the Aspin–Welch t-test. A value of *P* < 0.05 was considered statistically significant.

## Results

### General physical characteristics

Blood glucose levels, BWs, and bladder weights (BlaWs) are shown in Table 1. The blood glucose levels of DM rats (541.0 ± 25.3 mg/dL) were about 3-fold higher than those of normal rats (161.4 ± 7.6 mg/dL). Silodosin did not affect blood glucose levels. The BlaW of DM rats (0.220 ± 0.010 g) were approximately double that of normal rats (0.107 ± 0.005 g).

**Table 1:** Chronic effects of silodosin on blood glucose, BW, and BlaW in STZ-induced DM rats

Group	Blood glucose (mg/dL)	BW (g)	BlaW (g)
Normal	161.4 ± 7.6	278.5 ± 10.7	0.107 ± 0.005
DM-Vehicle	541.0 ± 25.3^†††^	260.4 ± 4.6	0.220 ± 0.010^†††^
DM-Silodosin 0.3 mg/kg/day	521.4 ± 15.2^†††^	262.5 ± 4.2	0.223 ± 0.014^†††^
DM-Silodosin 1.0 mg/kg/day	474.6 ± 27.5^†††^	271.8 ± 8.1	0.210 ± 0.014^†††^

Data are presented as means  ±  S.E.M (*n*  =  10–13). ^†††^ *P*  <  0.001 versus normal rats. BW, body weight; BlaW, bladder weight; DM, diabetes mellitus

### CMG study

**Fig. 1:**
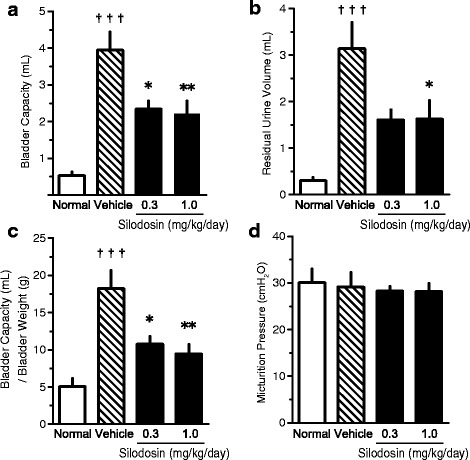
Effects of silodosin (0.3 and 1.0 mg/kg/day, s.c.) on bladder capacity (BC) (**a**), residual volume (RV) (**b**), BC/bladder weight (BlaW) (**c**), and intravesical pressure (IVP) (**d**) in rats on cystometrography. Data are presented as means  ±  S.E.M. (*n*  =  10–13). †††*P*  <  0.001 versus normal rats (Student’s *t*-test or Aspin–Welch *t*-test). *P  <  0.05, **P  <  0.01 versus diabetes mellitus (DM)-Vehicle rats (Dunnett’s test)

Cystometry performed in anesthetized DM rats indicated that BC and RV significantly increased 9 weeks after STZ injection, as compared to normal rats. In contrast, IVP did not differ between the DM rats and normal rats. In addition, the baseline pressure and threshold pressure did not differ between the DM rats and normal rats (data not shown). Silodosin significantly decreased BC and RV (Fig. 1A and B). In addition, the bladder extension (BC/BlaW ratio) increased in DM rats, indicating that the bladder had become thin (i.e., bladder hyperextension). Silodosin decreased the ratio almost to that observed in normal rats (Fig. 1C). Silodosin had no effect on IVP (Fig. 1D).

### BBF measurement

**Fig. 2:**
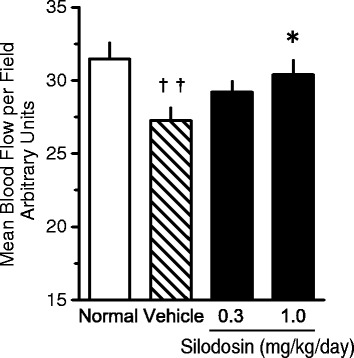
Effects of silodosin (0.3 and 1.0 mg/kg/day, s.c.) on bladder blood flow (BBF) in empty rat bladders. Data are presented as means  ±  S.E.M. (*n*  =  10–13). ††*P*  <  0.01 versus normal rats (Student’s *t*-test). *P  <  0.05 versus diabetes mellitus (DM)-Vehicle rats (Dunnett’s test)

A significant decrease in BBF in the empty bladder was observed in DM rats compared with normal rats. Silodosin at a dose of 1.0 mg/kg/day significantly suppressed the diabetes-induced decrease in BBF in the empty bladder (Fig. 2).

### Evaluation of nerve markers

**Fig. 3:**
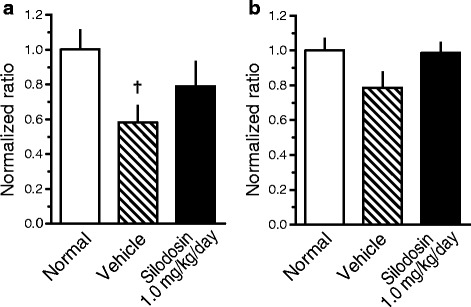
Gene expression of neurofilament-M (NF-M) (**a**) and peripherin (**b**) in the bladders of normal, diabetes mellitus (DM)-Vehicle, and DM-Silodosin rats. Values are shown as the ratio to the normal rats. Data are presented as means  ±  S.E.M. (*n*  =  6–8). †*P*  <  0.05 versus normal rats (Student’s *t*-test)

**Fig. 4:**
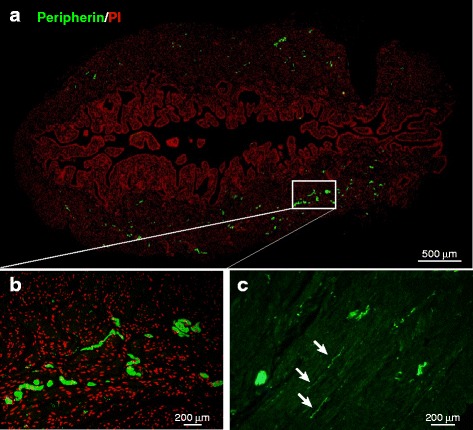
Representative immunofluorescence staining of peripherin-immunoreactive nerves (green) and propidium iodide-positive nuclei (red) in vertical sections of the bladder. **a** Whole bladder. **b** and **c**, Extended image. A few axons branched off from the plexus. A weaker b signal was observed under increased magnification

**Fig. 5:**
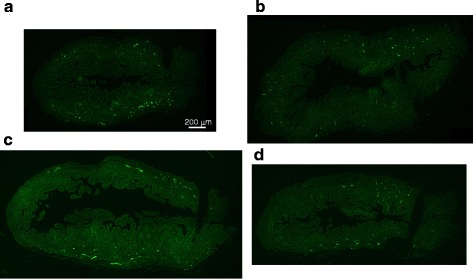
Peripherin expression in the vertical section of the whole bladder. Photomicrographs of labeled bladder sections were captured using a DP72 digital camera (Olympus, Tokyo, Japan) attached to an Olympus IX71 microscope. **a** Normal bladder. **b** Streptozotocin (STZ)-induced diabetes mellitus (DM)-Vehicle bladder. **c** and **d**, STZ-induced DM-chronic s.c. infusion of silodosin 0.3 and 1.0 mg/kg/day bladder, respectively

**Fig. 6:**
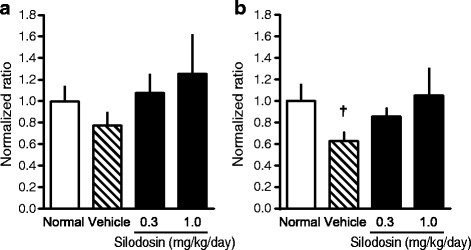
Immunohistochemical analysis of the effects of silodosin on neurofilament-M (NF-M) (**a**) and peripherin (**b**) in the bladder. The whole bladder was photographed and tiled with high accuracy using cellSens software. WinROOF was used to measure the total area of the photographed bladder and immunopositive bundles. Values are shown as the ratio to the normal rats. Data are presented as means  ±  S.E.M. (*n*  =  7–8). †*P*  <  0.05 versus normal rats (Student’s *t*-test)

We evaluated the expression of the neuron-specific markers NF-M (an intermediate filament that provides support for neuronal structure) and peripherin (a sensory neuron-specific marker) [23] by RT-PCR and immunohistochemistry. The mRNA expressions trended to decrease in DM rats and suppressed in silodosin-treated rats (Fig. 3A and B). Immunohistochemical analysis indicated that peripherin was distributed in the smooth muscular layer (low-power field) (Fig. 4A and B), and a weaker signal was observed at high magnification (Fig. 4C). These signals were weaker in full bladders in DM rats than in those in normal rats (Fig. 5). The signals in the silodosin group were similar to those in the normal group. Quantified signals are shown in Fig. 6. Signals for peripherin were significantly decreased in DM rats, and this decrease was suppressed by silodosin. Similar improvement in NF-M was observed on immunohistochemical analysis.

## Discussion

In this study, we examined the changes in bladder function and the effects of silodosin in STZ-induced DM rats. Nine weeks after STZ induction, urinary dysfunction was observed on a CMG. Increases in BC and RV were observed in the DM group, and the BC/BlaW ratio was high. Furthermore, BBF was decreased and both gene and protein expression levels of NF-M and peripherin, which are neuronal markers, were decreased in bladder tissue in the DM group. These results suggest that a decrease in BBF and bladder hyperextension with sensory disorder, i.e., UAB-like symptoms, occurred in the DM group. The alpha_1A_-AR antagonist silodosin showed inhibitory effects against all of these changes in the DM group. Silodosin shows preventive or curative effects against UAB-like symptoms in DM, and these effects may be caused by the improvement in the sensory disorder by preventing the decrease in BBF.

Autonomic nervous system disorders due to peripheral neuropathy, which is one of the 3 major complications of DM, are known to be responsible for urination disorders. LUTS have been reported in various DM rat models. The STZ-induced DM rats showed pathological progress from bladder irritation symptoms (compensatory response), such as frequent urination, to UAB symptoms (decompensatory response), such as increases in BC and RV [5, 7, 24]. In the CMG study, no change in IVP was observed in the DM group 9 weeks after STZ treatment, but increases in BC and RV were observed. We did not identify the reason IVP did not change, but we think that a longer treatment period may be required to observe the differences. In the present experiment, UAB-like symptoms were examined by the evaluation of RV and bladder hyperextension, although changes in IVP were not observed.

We used the BC/BlaW ratio as a new index to evaluate bladder extension in this study. The BC/BlaW ratio was high in the DM group compared with the normal group. Increases in BC and RV were also observed in the BOO rat model. However, there was little difference in the BC/BlaW ratio between the normal and BOO rats [25, 26]. As powerful contractions due to urethral obstruction are noted in the BOO rat model, the bladder smooth muscles probably became thick [27]. On the other hand, bladder sensation of DM rats decrease, and BC increase that is bladder smooth muscles became thin. Therefore, it is possible that the BC/BlaW ratio would be a useful index to quantitatively evaluate bladder extension accompanied with diminished bladder sensation.

As noted above, diminished bladder sensation is a symptom of peripheral neuropathy. The vascular supply in peripheral nerves is sparse and blood flow is likely to be compromised and lack autoregulation. The system causes peripheral nerves to be vulnerable to ischemia [28]. Therefore, it is suggested that peripheral neuropathy is closely related to decreased blood flow. In this study, BBF and the levels of neuronal markers, NF-M and peripherin, were measured in the bladder. NF-M and peripherin are intermediate filaments expressed in nerve cells, and peripherin is used as a specific marker of sensory neurons [23]. Therefore, measurement of peripherin expression in the bladder would be a useful index for evaluating bladder sensation. In this study, BBF decreased after 9 weeks of STZ treatment in DM rats. Although it was reported that blood vessel density decreased in DM rat bladders after 20 weeks of STZ treatment [8], ischemia was considered to have occurred after 9 weeks of STZ treatment because a decrease in BBF was observed at this time. Therefore, bladder nerve density was thought to have decreased, resulting in diminished bladder sensation.

We examined the effects of the alpha_1A_-AR antagonist silodosin on these changes in DM rats. Urinary function improved after chronic injection of silodosin for 8 weeks. In addition, silodosin inhibited the decrease in BBF and improved the decrease in the number of peripherin-positive sensory nerves in addition to NF-M-positive nerves.

The mechanism underlying the improvement in bladder dysfunction did not involve a decrease in blood glucose levels, as silodosin did not affect the blood glucose levels. Generally, poor contractility, detrusor sphincter dyssynergia, and decline of bladder sensation are considered to be the causes of RV increase and bladder hyperextension. At these concentrations, silodosin specifically inhibited alpha_1A_-AR but not alpha_1B_-AR or alpha_1D_–AR [29]. Silodosin shows urethral relaxation effect. But it was reported that urethral resistance is weaker in female than in male rats [30]. The BBF decrease is closely related to peripheral neuropathy, and alpha_1_-ARs are expressed in the bladder arteries. Furthermore, it has been reported that alpha_1_-AR antagonists increase BBF [18–20]. It may be possible that silodosin improved the decreased bladder sensation due to inhibition of ischemia by ameliorating the decrease in BBF in this DM rat model.

In the present study, silodosin treatment was initiated in the early stages of DM. Therefore, further studies are required to examine the curative effect of silodosin administered after DM progression. Furthermore, in this study, we did not perform comparisons with other alpha_1_-AR antagonists. A more detailed investigation is required to clarify the mechanism underlying the involvement of alpha_1_-AR subtypes in UAB by the comparison with other alpha_1_-AR antagonists with different alpha_1_-AR subtype selectivity.

## Conclusions

In conclusion, the alpha_1A_-AR antagonist silodosin showed a protective effect against impaired bladder function indicated by UAB-like symptoms characterized by increased RV and bladder hyperextension in STZ-induced DM rats.

## Abbreviations

AR: Adrenoceptor
BBF: Bladder blood flow
BC: Bladder capacity
BlaW: Bladder weight
BOO: Bladder outlet obstruction
BW: Body weight
CMG: Cystometrogram
DM: Diabetes mellitus
DUA: Detrusor underactivity
IVP: Intravesical pressure
LUTS: Lower urinary tract symptoms
MV: Micturition volume
NF-M: Neurofilament-M
OAB: Overactive bladder
RV: Residual volume
STZ: Streptozotocin
UAB: Underactive bladder
